# Airway cellularity, lipid laden macrophages and microbiology of gastric juice and airways in children with reflux oesophagitis

**DOI:** 10.1186/1465-9921-6-72

**Published:** 2005-07-15

**Authors:** AB Chang, NC Cox, J Purcell, JM Marchant, PJ Lewindon, GJ Cleghorn, LC Ee, GD Withers, MK Patrick, J Faoagali

**Affiliations:** 1 Department of Paediatrics, University of Queensland, Brisbane, Australia; 2 Department of Respiratory Medicine, Royal Children's Hospital, Brisbane, Australia; 3 Department of Gastroenterology, Royal Children's Hospital, Brisbane, Australia; 4 Department of Anatomical Pathology and Cytopathology, Queensland Health Pathology Service, Royal Brisbane Hospital, Brisbane, Australia; 5 Department of Microbiology, Queensland Health Pathology Service, Royal Brisbane Hospital, Brisbane, Australia

## Abstract

**Background:**

Gastroesophageal reflux disease (GORD) can cause respiratory disease in children from recurrent aspiration of gastric contents. GORD can be defined in several ways and one of the most common method is presence of reflux oesophagitis. In children with GORD and respiratory disease, airway neutrophilia has been described. However, there are no prospective studies that have examined airway cellularity in children with GORD but without respiratory disease. The aims of the study were to compare (1) BAL cellularity and lipid laden macrophage index (LLMI) and, (2) microbiology of BAL and gastric juices of children with GORD (G+) to those without (G-).

**Methods:**

In 150 children aged <14-years, gastric aspirates and bronchoscopic airway lavage (BAL) were obtained during elective flexible upper endoscopy. GORD was defined as presence of reflux oesophagitis on distal oesophageal biopsies.

**Results:**

BAL neutrophil% in G- group (n = 63) was marginally but significantly higher than that in the G+ group (n = 77), (median of 7.5 and 5 respectively, p = 0.002). Lipid laden macrophage index (LLMI), BAL percentages of lymphocyte, eosinophil and macrophage were similar between groups. Viral studies were negative in all, bacterial cultures positive in 20.7% of BALs and in 5.3% of gastric aspirates. BAL cultures did not reflect gastric aspirate cultures in all but one child.

**Conclusion:**

In children without respiratory disease, GORD defined by presence of reflux oesophagitis, is not associated with BAL cellular profile or LLMI abnormality. Abnormal microbiology of the airways, when present, is not related to reflux oesophagitis and does not reflect that of gastric juices.

## Introduction

Gastroesophageal reflux (GOR) is very common and defined as the passage of gastric contents into the oesophagus. GOR disease (GORD) is defined as symptoms or complications of GOR [1]. GORD includes the presence of oesophagitis, histologically defined on oesophageal biopsy, and increased reflux index on pHmetry in association with appropriate symptoms [2,1]. GOR and GORD is associated with pulmonary disease and postulated mechanisms include aspiration of gastric components, tracheo-gastric reflex, and sensory nerve stimulation [3]. Secondary aspiration relates to aspiration of gastric contents, which contain oral and ingested micro-organisms as well as gastric juices. With no gold standard of defining recurrent aspiration [4], current tests include nuclear medicine tests).)[5] and, quantification of lipid laden macrophages in bronchoalveolar lavage (BAL) fluid [6]. It is controversial whether an increased lipid laden macrophages index (LLMI) is a useful indicator for recurrent pulmonary aspiration [7,8]. Although LLMI is increased in aspiration lung disease, it is also found in other lung diseases [6,9,10]. Increased LLMI has been well documented in highly selected groups of children with lung disease [11] but there is little data regarding BAL of children without lung disease and GORD.

Assessment of airway profile is increasingly used in research as well as in clinical medicine for supportive (but not definitive) diagnosis of respiratory diseases in children [12,13]. The airway cellular profile of children with GORD without chronic lung disease is unknown and this knowledge will be useful for comparative clinical and research purposes.

The aims of the study were to compare, (1) BAL cellularity and lipid laden macrophage index (LLMI) and, (2) microbiology of BAL and gastric juices of children with GORD (G+) to those without (G-). We hypothesised that airways of children with reflux oesophagitis were more likely to have increased LLMI and neutrophilia from recurrent small volume aspiration, and that the bacterial flora in the lungs would be similar to that in the gastric aspirate fluid.

## Methods

Children aged 0.75–14 years undergoing elective flexible upper endoscopy were invited to participate in the study (August 2002 till June 2004). All children undergoing flexible upper endoscopy had seen a consultant paediatric gastroenterologist and the procedure performed under general anaesthesia including endotracheal intubation. Children were enrolled for the study on the morning of their procedure. Medical history was obtained from a parent on a standardised proforma for all children. Exclusion criteria were; children with neuro-developmental abnormalities, known underlying cardiorespiratory disease other then asthma and those with a clinical history of primary aspiration (coughs and chokes with feeds at least twice a week). GORD was considered present if histology of distal oesophageal biopsy showed reflux oesophagitis determined by pathologists blinded to the child's respiratory history [14]. Written consent was obtained and the study approved by our institution's human ethics committee.

At commencement of the flexible upper endoscopy, gastric juice was obtained directly under vision and suctioned into a mucus trap. When <0.5 mls was obtained, 5 or 10 mls of saline flush was used and colony count of an organism (if cultured) was corrected by the same factor. To obtain BAL fluid, a non-bronchoscopic standardised and repeatable [15]. technique was utilised. Briefly, with the child's head turned to the left, an 8F catheter was passed as far as possible through the endotracheal tube, ensuring that it went beyond the estimated carina site. Sterile normal saline (1 ml/kg to maximum of 20 mls) was instilled and suctioned into a mucus trap and this specimen was used for microbiology examination. A further 1 ml/kg (maximum of 20 mls) was instilled and the 2^nd ^collection utilised for cytology and lipid laden macrophage count. Cell count was performed on the cell suspension, cytocentrifuge slides were prepared and stained (modified Wright's stain, Diff Quik, Lab Aids, Narrabeen, NSW, Australia) for cell differential profile (400 cells counted when possible). Additional slides were prepared for LLMI using Oil Red O stain (Sigma Chemicals) where 100 macrophages counted and scored 0–4 [16,4]. LLMI (range 0–400) was obtained by the addition of these scores. All cellular and LLMI examinations were performed by cytologists blinded to the children's medical history.

Quantitative aerobic cultures of bacteria were undertaken on BAL and gastric juices using standard sterile loops (10 & 100 ul) on blood and chocolate agar plates for detection of aerobic bacteria. Plates were incubated at 35°C for 48-hours and isolates counted and identified to the genus level. Positive bacterial culture was defined as growth of ≥10^4 ^colony forming unit/ml [17]. Viral studies were also performed on BAL; direct immunofluorescence antigen (DFA) was used to detect RSV, adenovirus, parainfluenza viruses 1,2,3 and influenza A and B. If viral direct immunofluorescent antigen testing was negative, nucleic acid amplification (NAA) tests were undertaken for all the above viruses using multiplex PCR [18]

### Statistical analysis

Children were categorised having GORD (G+) and not having GORD (G-). Chi square was used to compare categorical variables between groups and odds ratio described. Data were not normally distributed and thus non parametric analyses were used; Mann-Whitney for comparisons between 2 groups and Kruskal Wallis when >2 groups were compared. Medians and inter-quartile range (IQR) were used for all descriptive data. Two tailed p value of <0.05 was considered significant. SPSS ver 11 was utilised for all statistical calculation.

## Results

Median age of the 150 children (91 boys and 56 girls) recruited was 8.2 years (IQR 7). The primary indications for oesophago-gastroscopy were abdominal pain (n = 77), recurrent vomiting (n = 35), poor weight gain (n = 20), review of previous lesion (n = 19) and choking (n = 17); some children had more than one primary indication for oesophago-gastroscopy. Most (n = 136, 90.7%) children were clinically suspected of having GORD and oesophagitis was present in 77 (51.3%) children. There were 77 children in G+ category, 73 in G-. Only 17 children had tobacco smoke exposure and as numbers were small, comparisons were not made.

G- group (median 7, IQR 13) had a significant but small increase in BAL neutrophil % when compared to the G+ group (5, 7.5), p = 0.002. There was no significant difference in percentages of macrophages, lymphocytes, eosinophils and LLMI in BAL between G+ and G- groups (table 1, p range 0.23 to 0.78). When children whose BAL showed positive bacterial culture were excluded (n = 31), BAL neutrophil % was still significantly higher in G- (n = 52) than G+ (n = 67) children, p = 0.009; median of 7 vs 4% respectively. Percentages of macrophages, lymphocytes, eosinophils and LLMI in BAL between G+ and G- groups remained not significantly different when those with BAL positive culture were excluded.

**Table 1:** Cellular profile of children grouped by presence and absence of GORD

	**G+ **N = 77	**G- **N = 73
Lymphocyte %		
Median, IQR	4.0, 3	4.4, 4
Neutrophil %		
Median, IQR	5, 7.5	7.5, 14
Macrophage %		
Median, IQR	89, 16	88.3, 14
Eosinophil %		
Median, IQR	0, 0	0, 0
Total cell count		
Median, IQR	96, 175	116, 83.5
LLM index		
Median, IQR	42, 47	38, 30

G+ = group with reflux oesophagitis

G- = group without reflux oesophagitis

Viral studies (DFA and NAA) were negative in all the BAL samples. Positive bacterial cultures was found in the BALs of 31 children (20.7%) and the gastric aspirates from 8 (5.3%) children, table 2. Eighteen children had a significant growth of *S. pneumoniae *in their BAL but only one of these children had significant growth of *S pneumoniae *in their gastric aspirate (10^5^). *S. aureus *was found in the BAL of 2 children and one of these also had a low count(10^2^) of S. aureus in their gastric aspirate. The cellular profile of these 31 children (median %neutrophils was 20, IQR 34; %lymphocytes 6, 8.5; %macrophages 65.5, 34) was significantly different to those without positive BAL culture (%neutrophils 5, 4; %lymphocytes 3, 4; %macrophages 90, 9); p of 0.00001 for all cell types. Children who were G+ were no more likely to have positive BAL culture than the G- group (table 3). Thirty seven children had recent use (within a week) of proton pump inhibitors (PPI). Use of PPI did not influence G+/G- status (p = 0.452) and also had no significant effect on BAL positive culture state (p = 0.762) or gastric aspirate culture (p = 0.092).

**Table 2:** Positive bacteria culture in BAL and gastric aspirate

Growth of organism of ≥10^4 ^colony forming unit	BAL n	Gastric aspirate n
*S. pneumoniae*	19	6
*H. influenzae*	10	0
*M. catarrhalis*	7	0
*S. aureus*	3	0
Candida	0	2

**Table 3:** Comparisons of groups with positive bacterial culture in BAL

	BAL culture			
Group category	Negative (<10^4 ^cfu/ml)	Positive (≥10^4 ^cfu/ml)	p value	OR, 95% CI
G-	52	17		
G+	67	14	0.27	0.64, 0.29–1.42

G+ = group with reflux oesophagitis

G- = group without reflux oesophagitis

## Discussion

In 150 children, we have shown that children with GORD and without an underlying lung problem have no abnormality in their airway cellular profile or LLMI. Indeed the percentage of neutrophils was significantly higher in the G- group than in the G+ group (the difference between the groups was however small and not clinically significant). We have also shown that positive bacterial culture with recognised respiratory pathogens was relatively high at 20.6% and was not influenced by G+ state. Lastly we showed that the microbiology of airways does not reflect that of gastric aspirates in children with and without GORD.

This is the first study that has examined airway cellularity and microbiology of airways of children without an underlying respiratory illness in relation with GORD. Although the percentage of neutrophils in BAL of G- children was higher than that in G+ children, the difference between the groups was small (median difference of 2%) and in the clinical context this is not significant. These BAL values are very close to the range described in normal children [12]. There is a paucity of data on airway findings in patients with GORD without an underlying lung disease. Our findings are similar to a small (n = 11) study in adults with GORD (without lung disease) which also described that GORD was not associated with airway neutrophilia. Our findings of absence of airway neutrophilia in children with GORD are in contrast to studies that have examined BAL in children with chronic respiratory disease [11]. It is likely that airway neutrophilia in the presence of respiratory illness is from the respiratory disease itself rather than from stimulation of the tracheo-gastric reflex. Indeed in our study, the percentage of neutrophils in BAL of children with GORD was lower than that in children without GORD. However, the absence of airway neutrophilia does not mean absence of neutrophilic inflammation as we did not examine for neutrophilic markers such as IL-8.

We did not find any difference in LLMI in children with or without GORD. In our study, LLMI is not a useful marker of presence of reflux oesophagitis in children without an underlying respiratory illness. The plausible explanations include; none of these children had secondary aspiration or/and LLMI is not a sensitive test for secondary aspiration. Indeed the utility of LLMI as a sensitive and specific marker of aspiration in children has been questioned [6,9,10]., and Colombo reported that LLMI was highest in patients underdoing chemotherapy and graft vs host disease [4]. Krishnan and colleagues recently described the poor specificity and sensitivity of LLMI for aspiration [19]. However they used tracheal aspirates[4], which is not representative of BAL [20,21]. BAL fluid is the common standardised method for examining airway cellularity in the respiratory field [12].

We found a high incidence of positive bacterial culture (of common respiratory bacteria in the BAL but not in gastric aspirate) in our cohort of children, defined on a chosen threshold of ≥10^4 ^cfu/ml based on previous studies [17]. However, the diagnostic threshold for quantitative culture on BAL for bronchitis in children (as opposed to pneumonia) is unknown and caution is needed in the interpretation of BAL microbiology [12]. As airway neutrophilia was also present in children with positive bacterial culture it is likely that culture results were significant. We did not find any relationship between airway and gastric aspirate microbiology, suggesting that aspiration of gastric pathogens was not significant in these children. Our findings are in support of a study showing that the stomach is not a source for colonization of the upper respiratory tract and pneumonia [22]. Swallowing of respiratory secretions of common respiratory bacteria is unlikely to result in positive gastric aspirate culture as gastric juice has a bactericidal effect; *S. aureus *is killed within 30–45 minutes of inoculation whereas *P. aeruginosa *is killed in 60–90 mins [23].

Our data may not be extrapolated to other definitions of GORD. We examined GORD defined by oesophageal biopsy, a common method of diagnosing GORD in children in Australia. In our institution, oesophago-gastroscopy is more commonly performed than pHmetry (approximately 800 and 300 respectively per year). A variety of techniques are utilised to confirm or support the diagnosis of GORD. However there is no single perfect method for the objective definition of all GORD types. Each modality has its advantages and disadvantages/limitations. Nevertheless, arguably oesophagitis is the gold standard definition but is also likely the least sensitive diagnostic method especially when GORD is mild and/or occasional or intermittent. The American Gastroenterology Association (AGA) guidelines on pHmetry stated "In the absence of oesophagitis, there is no gold standard for the definition of GERD..." [2]. pHmetry has been reported to be more sensitive but there is considerable disagreement what constitutes an abnormal pHmetry [1]. Also, some gastroenterologists argue that pHmetry alone cannot be used to diagnose GORD, given the known problems outlined in the AGA guidelines. Thus, similar findings using GORD based on pHmetry and perhaps multi-channel intraluminal electrical impedance monitoring, (arguably a more sensitive method for evaluating GORD variants ie acid and non-acid reflux [24]) would be necessary to conclude that all types of GORD are associated with normal airway cellular profile in otherwise well children. However, given that there was no significant abnormality in both groups of children examined in this study, it is unlikely that any type of acid related GORD will be associated with abnormal airway cellularity in children without lung disease.

## Conclusion

We conclude that, in children without respiratory disease, presence of GORD defined by reflux oesophagitis, is not associated with BAL cellular profile or LLMI abnormality. Abnormal microbiology of the airways, when present, does not reflect that of gastric juices and, is not associated with reflux oesophagitis.

## References

[B1] Rudolph CD, Mazur LJ, Liptak GS, Baker RD, Boyle JT, Colletti RB, Gerson WT, Werlin SL (2001). Guidelines for evaluation and treatment of gastroesophageal reflux in infants and children: recommendations of the North American Society for Pediatric Gastroenterology and Nutrition. J Pediatr Gastroenterol Nutr.

[B2] (1996). American Gastroenterological Association medical position statement: guidelines on the use of esophageal pH recording. Gastroenterology.

[B3] Daoui S, Agostino B, Gallelli L, Emonds X, Rossi F, Advenier C (2002). Tachykinins and airway microvascular leakage induced by HCl intra-oesophageal instillation. Eur Respir J.

[B4] Colombo JL (1999). Pulmonary aspiration and lipid-laden macrophages: In search of Gold (standards). Pediatr Pulmonol.

[B5] Bar-Sever Z, Connolly LP, Treves ST (1995). The radionuclide salivagram in children with pulmonary disease and a high risk of aspiration. Pediatr Radiol.

[B6] Bauer ML, Lyrene RK (1999). Chronic aspiration in children: evaluation of the lipid-laden macrophage index. Pediatr Pulmonol.

[B7] Colombo JL, Hallberg TK (1987). Recurrent aspiration in children: lipid-laden alveolar macrophage quantitation. Pediatr Pulmonol.

[B8] Lopez-Andreu JA, Roques-Serradilla JM, Cortell-Aznar I (2003). Fat-laden macrophages as a marker of reflux aspiration: still of some help. J Pediatr Gastroenterol Nutr.

[B9] Knauer-Fischer S, Ratjen F (1999). Lipid-laden macrophages in bronchoalveolar lavage fluid as a marker for pulmonary aspiration. Pediatr Pulmonol.

[B10] Kazachkov MY, Muhlebach MS, Livasy CA, Noah TL (2001). Lipid-laden macrophage index and inflammation in bronchoalveolar lavage fluids in children. Eur Respir J.

[B11] Sacco O, Fregonese B, Silvestri M, Sabatini F, Mattioli G, Rossi GA (2000). Bronchoalveolar lavage and esophageal pH monitoring data in children with "difficult to treat" respiratory symptoms. Pediatr Pulmonol.

[B12] de Blic J, Midulla F, Barbato A, Clement A, Dab I, Eber E, Green C, Grigg J, Kotecha S, Kurland G, Pohunek P, Ratjen F, Rossi G (2000). Bronchoalveolar lavage in children. ERS Task Force on bronchoalveolar lavage in children. European Respiratory Society. Eur Respir J.

[B13] Riedler J, Grigg J, Robertson CF (1995). Role of bronchoalveolar lavage in children with lung disease. Eur Respir J.

[B14] Yellon RF, Coticchia J, Dixit S Esophageal biopsy for the diagnosis of gastroesophageal reflux-associated otolaryngologic problems in children. The American Journal of Medicine.

[B15] Warke TJ, Kamath S, Fitch PS, Brown V, Shields MD, Ennis M (2001). The repeatability of nonbronchoscopic bronchoalveolar lavage differential cell counts. Eur Respir J.

[B16] Corwin RW, Irwin RS (1985). The lipid-laden alveolar macrophage as a marker of aspiration in parenchymal lung disease. Am Rev Respir Dis.

[B17] Labenne M, Poyart C, Rambaud C, Goldfarb B, Pron B, Jouvet P, Delamare C, Sebag G, Hubert P (1999). Blind protected specimen brush and bronchoalveolar lavage in ventilated children. Crit Care Med.

[B18] Syrmis MW, Whiley DM, Thomas M, Mackay IM, Williamson J, Siebert DJ, Nissen MD, Sloots TP (2004). A sensitive, specific, and cost-effective multiplex reverse transcriptase-PCR assay for the detection of seven common respiratory viruses in respiratory samples. J Mol Diagn.

[B19] Krishnan U, Mitchell JD, Tobias V, Day AS, Bohane TD (2002). Fat laden macrophages in tracheal aspirates as a marker of reflux aspiration: a negative report. J Pediatr Gastroenterol Nutr.

[B20] Malikides N, Hughes KJ, Hodgson DR, Hodgson JL (2003). Comparison of tracheal aspirates and bronchoalveolar lavage in racehorses. 2. Evaluation of the diagnostic significance of neutrophil percentage. Aust Vet J.

[B21] Mentec H, May-Michelangeli L, Rabbat A, Varon E, Le Turdu F, Bleichner G (2004). Blind and bronchoscopic sampling methods in suspected ventilator-associated pneumonia. A multicentre prospective study. Intensive Care Med.

[B22] Bonten MJ, Gaillard CA, van Tiel FH, Smeets HG, van der GS, Stobberingh EE (1994). The stomach is not a source for colonization of the upper respiratory tract and pneumonia in ICU patients. Chest.

[B23] Williams C (2001). Occurrence and significance of gastric colonization during acid-inhibitory therapy. Best Pract Res Clin Gastroenterol.

[B24] Shay S, Tutuian R, Sifrim D, Vela M, Wise J, Balaji N, Zhang X, Adhami T, Murray J, Peters J, Castell D (2004). Twenty-four hour ambulatory simultaneous impedance and pH monitoring: a multicenter report of normal values from 60 healthy volunteers. Am J Gastroenterol.

